# Cellulolytic enzyme expression and simultaneous conversion of lignocellulosic sugars into ethanol and xylitol by a new *Candida tropicalis* strain

**DOI:** 10.1186/s13068-016-0575-1

**Published:** 2016-07-26

**Authors:** Anu Jose Mattam, Arindam Kuila, Niranjan Suralikerimath, Nettem Choudary, Peddy V. C. Rao, Harshad Ravindra Velankar

**Affiliations:** Bioprocess Group, Hindustan Petroleum Corporation Limited, HP Green R&D Centre, KIADB Industrial Area, Tarabahalli, Devanagundi, Hoskote, Bengaluru, 560067 India

**Keywords:** *Candida tropicalis*, Cellulase, Ethanol, Wheat straw, Xylanase, Xylitol

## Abstract

**Background:**

Lignocellulosic ethanol production involves major steps such as thermochemical pretreatment of biomass, enzymatic hydrolysis of pre-treated biomass and the fermentation of released sugars into ethanol. At least two different organisms are conventionally utilized for producing cellulolytic enzymes and for ethanol production through fermentation, whereas in the present study a single yeast isolate with the capacity to simultaneously produce cellulases and xylanases and ferment the released sugars into ethanol and xylitol has been described.

**Results:**

A yeast strain isolated from soil samples and identified as *Candida tropicalis* MTCC 25057 expressed cellulases and xylanases over a wide range of temperatures (32 and 42 °C) and in the presence of different cellulosic substrates [carboxymethylcellulose and wheat straw (WS)]. The studies indicated that the cultivation of yeast at 42 °C in pre-treated hydrolysate containing 0.5 % WS resulted in proportional expression of cellulases (exoglucanases and endoglucanases) at concentrations of 114.1 and 97.8 U g^−1^ ds, respectively. A high xylanase activity (689.3 U g^−1^ ds) was also exhibited by the yeast under similar growth conditions. Maximum expression of cellulolytic enzymes by the yeast occurred within 24 h of incubation. Of the sugars released from biomass after pretreatment, 49 g L^−1^ xylose was aerobically converted into 15.8 g L^−1^ of xylitol. In addition, 25.4 g L^−1^ glucose released after the enzymatic hydrolysis of biomass was fermented by the same yeast to obtain an ethanol titer of 7.3 g L^−1^.

**Conclusions:**

During the present study, a new strain of *C. tropicalis* was isolated and found to have potential for consolidated bioprocessing (CBP) applications. The strain could grow in a wide range of process conditions (temperature, pH) and in the presence of lignocellulosic inhibitors such as furfural, HMF and acetic acid. The new yeast produced cellulolytic enzymes over a wide temperature range and in the presence of various cellulosic substrates. The cellulolytic enzymes produced by the yeast were effectively used for the hydrolysis of pretreated biomass. The released sugars, xylose and glucose were, respectively, converted into xylitol and ethanol. The potential shown by the new inhibitor tolerant cellulolytic *C. tropicalis* to produce ethanol or xylitol is of great industrial significance.

## Background

Lignocellulosic biofuels are promising alternatives to fossil fuels due to their less polluting nature, renewability and miscibility with conventional transportation fuels. The production of biofuels from biomass comprises a multistep process consisting of unit operations such as biomass pretreatment, enzymatic hydrolysis, fermentation and product separation. During the past two decades, significant research efforts have gone into the optimization and integration of each of these processes using a wide variety of biomass and biocatalysts [1]. The information generated through earlier studies has aided in setting up pilot scale facilities for lignocellulosic biofuel production [2].

Notwithstanding the advancements, lignocellulosic biofuel production technologies have not been widely commercialized. The cost of producing ethanol from lignocellulosic biomass is still considered to be higher than from sugar-based feedstock mainly because the cost of processing the biomass is substantial. To minimize the risks, biomass pretreatment processes are usually designed to take in multiple or mixed feedstock which adds up to the cost of its processing [3, 4]. The cost of biomass processing can, however, be controlled by selecting a process which is rapid, requires minimum catalyst (chemicals/enzymes) quantities as well as energy inputs, maximizes catalyst reuse, and results in minimal sugar degradation and inhibitor formation to achieve maximum sugar release through biomass depolymerisation [4–8].

The sugars released from biomass at different stages mainly consist of pentose and hexose monomers. Several industrial strains such as *Saccharomyces* sp. can convert hexose sugars into ethanol; albeit xylose conversion still remains challenging [9]. Although several wild-type microorganisms are known to convert pentoses into ethanol [10–12], a robust biocatalyst that can be utilized at the industrial levels is yet to be found. Studies from the recent past indicate the development of engineered ‘pentose-to-ethanol’ converting biocatalysts which may now become the way forward. As an alternative, the xylose sugars generated during biomass hydrolysis can also be fermented into a product with higher value than ethanol such as xylitol, whose co-production will reduce the cost of cellulosic ethanol production [6, 13].

Another projected approach to lower the cost of lignocellulosic ethanol production is via consolidated bioprocessing (CBP) with a single microorganism which can produce cellulolytic enzymes for biomass hydrolysis and can ferment the released sugars into ethanol, all together in a single step. However, CBP for cellulosic ethanol production at even the pilot scale remains largely hypothetical mainly due to inherent problems of suboptimal cellulase/xylanase expression or impaired fermentation yields [14]. Due to these reasons, microorganisms expressing cellulolytic enzymes are still not considered as a suitable candidate for carrying out ethanol fermentations and vice versa.

In the present study, a new isolate identified as *Candida tropicalis* was found to express cellulases and xylanases and convert xylose into xylitol and glucose into ethanol. Preliminary studies were carried out to determine the effects of substrates on cellulase and xylanase production by the yeast. The cellulolytic enzymes produced by the yeast isolate were used for the hydrolysis of pre-treated biomass and sugar hydrolysate was fermented into ethanol. The yeast also showed the ability to aerobically convert xylose into xylitol in the presence of inhibitory compounds. The new yeast isolate is a potential candidate for producing cellulosic ethanol via CBP.

## Results and discussion

A single microorganism that expresses cellulolytic enzymes for hydrolysis of biomass and ferments the released sugars into ethanol and other value added products such as xylitol is of potential interest to the industry. In this study, a new cellulolytic strain was isolated on an agar plate having CMC as the only carbon source. Microscopic examination indicated that the isolate was a yeast and 18S rRNA sequencing subsequently confirmed that it was a *C. tropicalis* strain. Since there are hardly any reports about cellulolytic *Candida* sp. the strain identity was reconfirmed by sequencing the variable D1–D2 domain of the 26S rRNA and the internal transcribed spacer (ITS) region of the 5.8S rRNA, as these have been projected as more authentic markers for strain identification than 18S rRNA [15, 16]. All the three sequences of MTCC 25057 showed a complete 100 % homology to *C. tropicalis*.

The cellulolytic enzymes produced by the new yeast isolate were used for the hydrolysis of pre-treated wheat straw and the released glucose was converted into ethanol by the same yeast strain. After acid pretreatment of wheat straw, the xylose generated in the hydrolysate could be converted into xylitol or used for producing cellulolytic enzymes. Barring an earlier report related to endoglucanase activities in *C. tropicalis* cultures after prolonged incubation periods [17] and despite the knowledge about the existence of genes coding for cellulolytic enzymes [GenBank:3636270; 3637086; 8296755, etc.], no other related reports were found.

### Tolerance of *Candida tropicalis* MTCC 25057 to inhibitory compounds

**Fig. 1:**
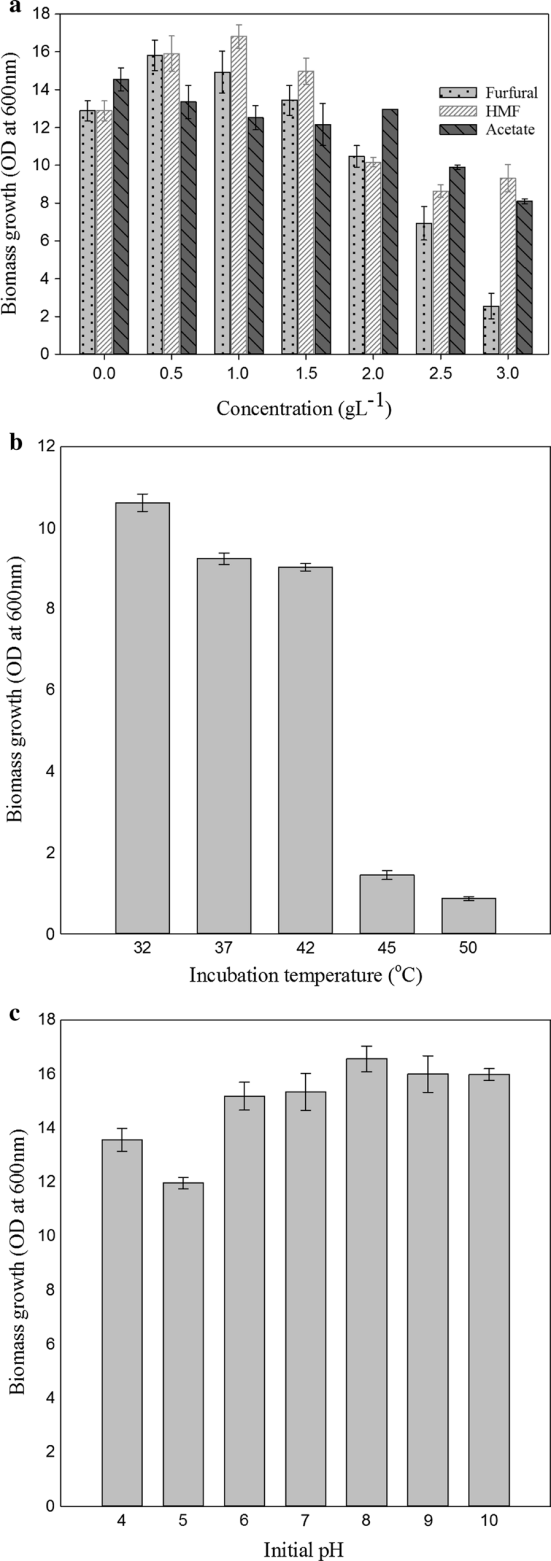
Effect of **a** different inhibitors (furfural, HMF, acetate) concentration, **b** temperature and **c** initial pH on growth of *Candida tropicalis.* The *error bar* range represents standard deviation from mean, *t* test significance level *p* < 0.005

For utilizing sugars in lignocellulosic hydrolysates, the biocatalyst has to withstand the presence of inhibitory compounds such as furfural, HMF and acetic acid that are usually generated during biomass pretreatment [18]. In the present study, wheat straw procured from local sources was subjected to pretreatment with 0.2 % (v/v) phosphoric acid as optimized previously in the authors’ laboratory [19]. The lignocellulosic hydrolysate contained glucose (~0.5 % w/v) and xylose (~1.5 % w/v) as the major components along with negligible quantities of inhibitory compounds such as furfural and HMF (~0.01 % w/v). Several reports indicate the formation of acetic acid during biomass pretreatment, although the same was not detected during our studies. Preliminary studies for determining the tolerance of the new yeast to higher concentrations of furfural, HMF as well as acetic acid, which might get generated during harsher pretreatments indicated that the *Candida* isolate tolerated furfural, HMF and acetate concentrations of 1.5, 2 and 2 g L^−1^, respectively (Fig. 1a). Further increase in the levels of furfural, HMF and acetic acid resulted in a gradual lowering of biomass densities (Fig. 1a). A recent report related to the fermentative capacities of *C. tropicalis* isolate to produce ethanol and xylitol in hydrolysate, indicated that the yeast could not function beyond furfural, HMF and acetate levels higher than 0.23, 0.15 and 1.37 g L^−1^ respectively [20]. In comparison, the new yeast isolate under investigation was found to be more tolerant to commonly occurring lignocellulosic inhibitors.

In addition to ethanol toxicity, osmotic stress and pH variations [21, 22], commercial scale ethanol bioprocesses also encounter increased temperatures during summer seasons during which the yeast is supposed to perform as normally as under optimal conditions [23]. We observed during our studies that cell growth of the new yeast isolate was not affected over a wide range of temperatures (32–42 °C) and pH (4–10) (Fig. 1b, c). Also, the cellulase and xylanase production by the yeast isolate remained largely unaffected over a wide range of temperatures (32–42 °C).

### Cellulase and xylanase production by *Candida tropicalis* MTCC 25057

**Fig. 2:**
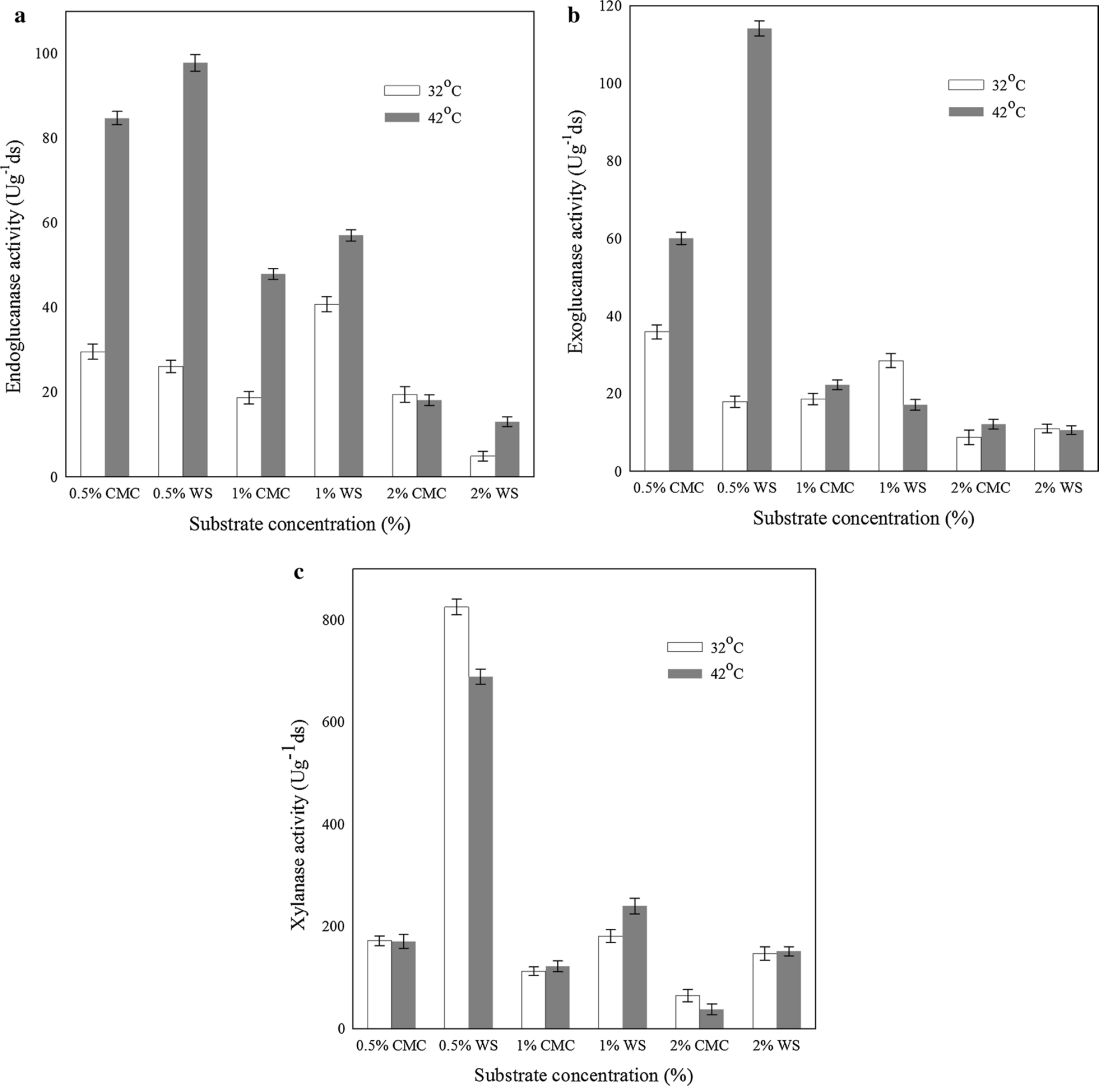
Effect of different substrates (CMC and Wheat Straw-WS) concentrations (0.5, 1 and 2 %) on **a** endoglucanase activity, **b** exoglucanase activity and **c** xylanase activity at 32 and 42 °C. The *error bar* range represents standard deviation from mean, *t*-test significance level *p* < 0.005

Earlier reports indicate that the temperature of incubation and the nature of substrates mainly affect cellulolytic enzyme production [24, 25]. Experiments were designed to determine cellulase (endoglucanase and exoglucanase) and xylanase production by the new yeast strain at different temperatures (32 and 42 °C), cellulosic substrates (CMC and WS) and at different concentrations (w/v) of 0.5, 1 and 2 % (Fig. 2) in pretreated lignocellulosic hydrolysate. Maximum endoglucanase activity of 98 ± 2 U g^−1^ ds was obtained using 0.5 % WS at 42 °C (Fig. 2a). Similarly, the exoglucanase production in hydrolysate containing 0.5 % wheat straw was maximum (114 ± 3 U g^−1^ ds) at 42 °C (Fig. 2b). At lower incubation temperature of 32 °C, the endo and exoglucanase levels in hydrolysate were lower than their corresponding levels at 42 °C with both the substrates and at all the concentrations tested (Fig. 2a, b).

Earlier studies have indicated that cellulolytic enzyme blends with approximately equal exoglucanase and endoglucanase activities are more suitable for optimal biomass hydrolysis [26]. In this work, we observed that amongst the two temperatures tested, maximum endoglucanase (Fig. 2a) and exoglucanase (Fig. 2b) activities were obtained at 42 °C, while 0.5 % WS was found to be a more suitable cellulosic substrate. The ratios of endo and exoglucanases at 42 °C were proportional and their activities were comparable to the previously reported cellulase expression levels by well-known enzyme producer strains such as *Trichoderma* and *Aspergillus* sp. [27–29].

Regarding xylanases, the activity obtained at 32 °C was marginally higher (825 ± 14 U g^−1^ ds) than that obtained at 42 °C (689 ± 17 U g^−1^ ds) with 0.5 % WS (Fig. 2c) in hydrolysate. The expression of xylanases with wheat straw could be attributed to the presence of xylan [30]; however, it was interesting to find xylanase production even in the presence of CMC, which is apparently xylan free. Earlier reports related to xylanase production indicate that the strains of *Aspergillus* sp.*, Penicillium* sp., etc. show very high xylanase activities ranging from 5000 to 7000 U g^−1^ ds [31, 32] along with cellulase production. It is expected that genetic improvements in the new yeast isolate may lead to increased expression of cellulolytic enzymes.

### Wheat straw hydrolysis

Different acids have been used to carry out biomass pretreatments [33, 34] and the use of phosphoric acid is known to generate lesser inhibitory compounds in the hydrolysate [35]. The quantity of inhibitory compounds generated during biomass pretreatment is mainly dependant on pretreatment severity. An earlier study from the author’s laboratory optimized wheat straw pretreatment using 0.2 % phosphoric acid at 150 °C for 15 min [19] and the same conditions were used for biomass pretreatment in the present study. The cellulose, hemicellulose and lignin content of biomass before and after phosphoric acid pretreatment was determined (Table 1). Phosphoric acid pretreatment of wheat straw caused approx. 65 % lignin removal and 64 % hemicellulose hydrolysis. After separating the hydrolysate and neutralizing the biomass, the cellulosic biomass was subjected to hydrolysis with the enzymes produced by the new yeast at an approximate enzyme loading of 20 U g^−1^ of wheat straw for 48 h at 50 °C. The sugar hydrolysate generated contained about 500 mg glucose and 30 mg xylose per gram of dry wheat straw used.

**Table 1:** Compositional analysis of wheat straw (before and after) treatment with 0.2 % (v/v) phosphoric acid at 150 °C for 15 min

	Cellulose (%)	Hemicellulose (%)	Lignin (%)
Before pretreatment	39.65 ± 2.1	7.95 ± 0.6	15.19 ± 1.2
After pretreatment	48.68 ± 1.8	2.85 ± 0.4	5.28 ± 0.8

It is widely acknowledged that the cost of producing cellulosic ethanol can be lowered by optimizing conversion of the released pentoses and hexoses into ethanol or by converting pentoses into an additional high value compound such as xylitol [36]. On the basis of earlier reports related to ethanol and xylitol production by *Candida* species [20, 36], investigations were carried out to determine ethanol and xylitol fermentation capacities of the new yeast isolate.

### Pentose and hexose fermentation by *Candida tropicalis* MTCC 25057 in synthetic media

Biomass consists of approx. 20–40 % of hemicellulose [37], which upon hydrolysis, releases xylose as the main component. The naturally occurring industrial ethanol producing yeast, *Saccharomyces cerevisae*, cannot utilize xylose and hence a hexose and pentose fermenting microorganism is needed. *Candida* sp. is a distinctive class of yeast that can ferment glucose into ethanol and xylose into xylitol and ethanol [20, 36–38]. Over the years, studies have shown that different strains of *Candida* sp. produce optimum levels of xylitol (from xylose) and ethanol (from glucose and xylose) under aerobic and anaerobic conditions, respectively [20].

**Fig. 3:**
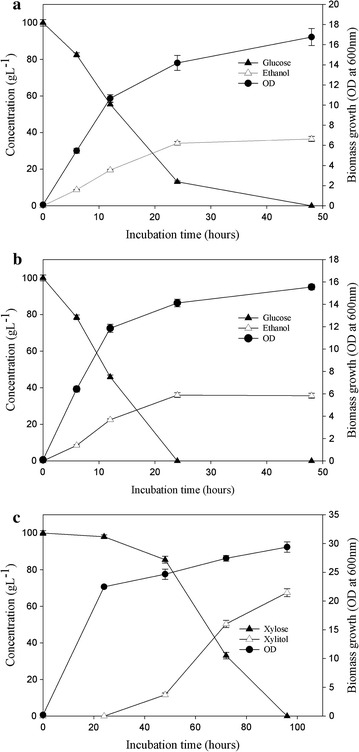
Fermentation of glucose under aerobic **a**, anaerobic (**b**) and of xylose under aerobic **c** conditions by *Candida tropicalis* at 32 °C in synthetic media. The *error bar* range represents standard deviation from mean, *t*-test significance level *p* < 0.005

During the current investigations, it was observed that *Candida* cell growth and ethanol production patterns were similar under both, aerobic and anaerobic conditions (Fig. 3a, b). Ethanol production using *Candida* strains is essentially an anaerobic process. However, the new *Candida* isolate could produce comparable concentrations of ethanol under aerobic conditions although the productivities were decreased.

On culturing the yeast strain in synthetic YEPD media under anaerobic conditions, maximum ethanol levels of ~36 g L^−1^ were obtained within 24 h, whereas similar ethanol concentrations were attained under aerobic conditions after 48 h of incubation (Fig. 3a, b). Under anaerobic and aerobic conditions, the corresponding glucose consumption of 100 and 80 % was observed within 24 h. Under aerobic conditions, the complete consumption of the remaining 20 % glucose required extended incubation for up to 48 h (Fig. 3a). Therefore, the ethanol yields (0.36 g g^−1^, i.e., ~72 % conversion) were similar while the ethanol productivities under anaerobic conditions (1.5 g L^−1^ h^−1^) were higher than under aerobic conditions (0.75 g L^−1^ h^−1^). Under both the conditions, approx. 3–4 g L^−1^ glycerol was detected in the medium (data not shown).

The *Candida tropicalis* isolate could also utilize xylose under aerobic conditions and produce xylitol (68 ± 2 g L^−1^) within 96 h of fermentation (Fig. 3c) when cultured in YEPD media. The xylitol yields and productivities obtained during aerobic fermentations were 0.67 g g^−1^ (67 % conversion) and 0.70 g L^−1^ h^−1^, respectively. No consumption of xylose was observed under anaerobic conditions even after extended incubation up to 120 h (data not shown). It has been previously reported that nicotinamide adenine dinucleotide phosphate (NADPH) is required for the functioning of xylose reductase, a key enzyme involved in xylitol production [39], which is regenerated only under aerobic conditions [40] possibly preventing anaerobic utilization of xylose.

**Fig. 4:**
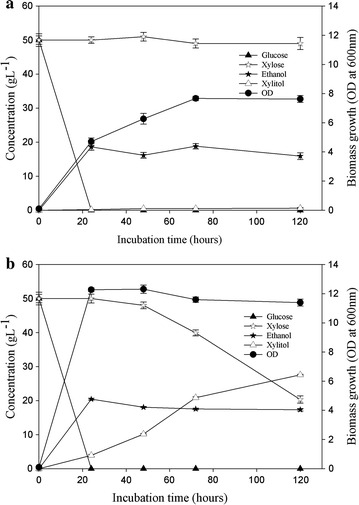
Fermentation of synthetic media containing glucose and xylose under **a** anaerobic and **b** aerobic conditions to produce ethanol and xylitol by *Candida tropicalis* at 32 °C. The* error bar* range represents standard deviation from mean, *t*-test significance level *p* < 0.005

Since lignocellulosic hydrolysate contains a mixture of pentose and hexose sugars, it was necessary to determine the ability of the isolate to ferment both simultaneously. Therefore, in order to simulate fermentation of lignocellulosic hydrolysate containing mixed sugars (hexose and pentose), the strain of *C. tropicalis* was cultured in synthetic lab media in which 50 g L^−1^ each of xylose and glucose had been added. The anaerobic cultivation of *C. tropicalis* in glucose–xylose medium led to complete utilization of glucose within 24 h whereas xylose remained unutilized in the medium (Fig. 4a). The maximum ethanol concentrations obtained under anaerobic (18.8 ± 0.8 g L^−1^) and aerobic (20.4 ± 1.3 g L^−1^) conditions as well as the ethanol yields of 0.37 and 0.4 g g^−1^ were found to be similar. However, no xylitol was detected during anaerobic fermentations of *Candida tropicalis* even till the end of the fermentation (Fig. 4a). Under aerobic cultivation, xylose utilization started 48 h after complete utilization of glucose (Fig. 4b) and a xylitol yield and productivity of 0.93 g g^−1^ and 0.28 g L^−1^ h^−1^, respectively, were obtained. The delayed onset of xylose utilization could be attributed either to the higher affinity of the xylose transporter for glucose or the repression of the first two genes of the xylose utilization pathway, viz. xylose reductase and xylitol dehydrogenase, as was previously reported for *Candida shehatae* and *Pichia stipitis* [41, 42]. The xylitol yield obtained in case of mixed sugar fermentation in this study was higher than the previously reported values in the range of 0.49–0.84 g g^−1^ produced by other *Candida strains* [43, 44]. Another important observation made during the studies was that the yield of xylitol obtained when xylose was the sole carbon source was lower (0.67 g g^−1^) than the yield (0.93 g g^−1^) obtained on using xylose and glucose in the media. The increased xylitol yield in the presence of mixed sugars might have occurred due to the utilization of glucose for biomass formation resulting in xylose being consumed only for xylitol formation and not biomass, or the sufficient availability of NADPH (essential for the optimal activity of the xylose reductase enzyme) which is regenerated best in the presence of glucose [40].

### Pentose and Hexose fermentation by *Candida tropicalis* MTCC 25057 in hydrolysate

**Fig. 5:**
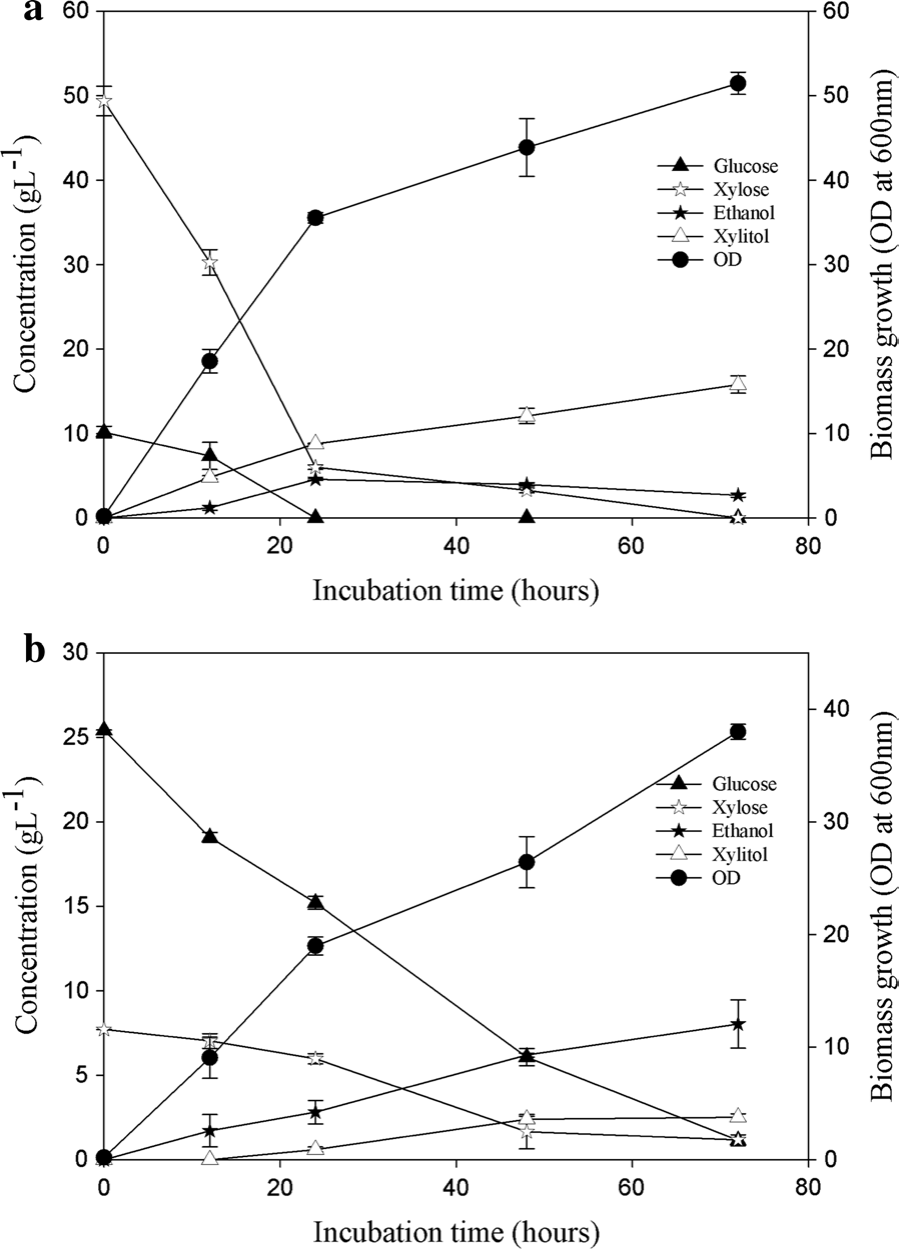
Fermentation of pretreated hydrolysate under anaerobic **a** and aerobic **b** conditions to produce ethanol and xylitol by *Candida tropicalis* at 32 °C. The* error bar* range represents standard deviation from mean, *t*-test significance level *p* < 0.005

Although wheat straw pretreatment with 0.2 % phosphoric acid has been optimized in our laboratory due to minimal inhibitor generation, here we have used harsher pretreatment conditions (4 % phosphoric acid) so as to achieve maximal xylose generation in the hydrolysate as this yeast isolate has the capability to withstand high inhibitor concentrations. Interestingly, even after the use of such highly acidic conditions for pretreatment, the amount of inhibitors produced was minimal—1 g L^−1^ furfural and 0.075 g L^−1^ HMF, reaffirming the use of phosphoric acid as an effective yet gentle pretreatment agent. The pretreated hydrolysate generated was fermented initially using the new yeast isolate after supplementation with minimal salts only. However, no ethanol or xylitol production was observed (data not shown). Subsequently, to meet the nutritional deficiencies, an additional nitrogen source (1 % yeast extract) was added to the hydrolysate which resulted in increased xylose utilization. Almost 60 % of the xylose was consumed within the first twelve hours of fermentation itself and no residual xylose was observed beyond 72 h of fermentation (Fig. 5a). Xylitol production reached maximum levels of ~15 ± 1 g L^−1^ in 72 h with an average yield of ~32 %. The hydrolysate also contained some amount of glucose (10 g L^−1^) which was converted into ethanol (~4.5 g L^−1^) in the initial 24 h.

In a similar study reported earlier, fermentation of concentrated corn cob hydrolysate (without nutritional supplementation) using a hydrolysate adapted *Candida* strain resulted in a xylitol yield of approx. 14.81 % with most of the xylose being diverted for biomass formation which affected the final yields [45]. In our study, a xylitol yield of ~32 % was achieved using a wild-type *Candida* isolate; although here too, most of the sugar was being used for cell growth instead of xylitol production. Further improvements in xylitol titers and yields may be achieved by strain adaptation, optimization of the cultivation conditions, enriching the lignocellulosic hydrolysate for better cell growth, etc.

The sugar hydrolysate obtained post-enzymatic saccharification of the pretreated wheat straw was also fermented using the same yeast strain. This hydrolysate has more hexoses than pentoses as it is derived from the cellulose rich biomass. In this case also, supplementation of the hydrolysate with additional N source led to greater diversion of the sugars to cell growth than to product formation. The yeast was able to convert ~25 g L^−1^ glucose to 7.3 ± 1.1 g L^−1^ ethanol with a net yield of 0.28 g g^−1^ (Fig. 5b), which was slightly lower than the ethanol yield observed in case of mixed sugar fermentation in synthetic medium. The sugar hydrolysate also contained ~7.5 g L^−1^ of xylose that was converted to xylitol in 24 h with a yield of 0.31 g g^−1^. The ethanol yield and productivity could be enhanced further by fine tuning the addition of nutritional supplements so as to ensure maximum sugar fermentation into ethanol production. A recently published review by Jouzani and Taherzadeh [46] comprehensively covers almost all the wild-type single microbial strains, microbial co-cultures as well as genetically engineered microorganisms with cellulolytic activities and ethanol producing abilities that showed potential for converting biomass into bioethanol via CBP. In comparison, the strain isolated in the present study also showed cellulolytic as well as ethanol fermenting abilities. In addition, the yeast strain also exhibited significant inhibitor tolerance and produced xylitol from hemicellulosic sugars. To establish the strain’s potential for CBP applications, detailed investigations into the metabolic pathways are needed.

Generally, ethanol producing yeast such as *Saccharomyces*, *Kluyveromyces* naturally lack cellulase expression and hence cellulolytic fungi such as *Trichoderma* and *Aspergillus* are used for producing cellulolytic enzymes. However, yeast cells are widely preferred as the hosts for recombinant enzyme production due to their robust nature, rapid growth rates and higher protein secretion [47–49]. In view of the advantages offered by yeast cells, research efforts have focussed on imparting cellulolytic abilities for their development as CBP-suitable hosts [50–52]. To develop cellulolytic yeast, multiple genes coding for various cellulases have to be cloned and their gene products need to be expressed in a stable manner, at a certain proportion and in higher quantities for achieving hydrolytic action on cellulose in a synergistic manner. The insertion of *δ*-retrotransposons regulates simultaneous expression of multiple genes [53, 54], whereas the synthetic promoter and terminator sequences improve the stability as well as activity of the mRNA [55, 56] while hybrid transcriptional activators enhance the secretion of cellulolytic enzymes [57]. Earlier studies have also focussed on increasing the extracellular enzyme secretion by manipulating the expression of proteins such as SNAREs, karyopherins and superoxide dismutase, which have been implicated in the regulation of intracellular membrane transport [58, 59]. However, all these approaches in yeast engineering have met with limited success since the cellulolytic enzyme yields were much lower than those produced by the established strains such as *Aspergillus*, *Trichoderma* etc. [60–62].

An ideal CBP operation for lignocellulosic bioethanol production would depend upon higher expression of hydrolytic enzymes for optimum biomass hydrolysis and the rapid fermentation of released sugars, all occurring within a relatively narrow range of process parameters. In actuality, since the temperature optima for enzyme production (30–32 °C), biomass hydrolysis (50 °C) and fermentation (30–32 °C) vary, the processes are still carried out in a successive manner. During our studies, the inhibitor tolerant strain of *Candida* exhibited maximal enzyme production (42 °C and pH 6–7) and biomass hydrolysis (at 50 °C and pH 5) within a relatively narrower range of process parameters, thereby indicating its potential for CBP applications.

Further improvements in the new yeast isolate can include overexpressing native cellulases by inserting multiple gene copies and the development of recombinants by inserting genes from conventional enzyme producers such as *Aspergillus* or *Trichoderma*. The thermostability, substrate specificity, etc. of native cellulases produced by *C. tropicalis* isolate can also be improved via protein engineering. Ongoing investigations in the authors’ laboratory also focus upon adaptation of the yeast to nutritionally deficient environments such as hydrolysate as well as imparting the ability to co-utilize hexose and pentose sugars for increased productivity during mixed sugar fermentations.

## Conclusions

Till now, several wild-type bacteria and filamentous fungi along with their recombinant strains and a few genetically engineered yeast were considered as potential CBP candidates for lignocellulosic ethanol production. The current work is the first definitive report describing a new strain of *Candida tropicalis* with ability to produce cellulolytic enzymes, ethanol and xylitol, thereby making it a prospective candidate for consolidated bioprocessing. The new yeast isolate also exhibited high tolerance to inhibitors such as furfural, HMF and acetic acid, found commonly in lignocellulosic hydrolysate. In addition, it produced cellulases and xylanases over a wide range of temperatures and in the presence of diverse cellulosic substrates. However, in order to carry out efficient hydrolysis of pre-treated biomass, the expression and activities of the cellulolytic enzymes will have to be significantly improved by the over-expression of heterologous cellulases and accessory proteins. Also, the ethanol and xylitol titers obtained during fermentations in hydrolysates were relatively lower despite the strain’s tolerance to inhibitors and this could be attributed to the existing nutritional deficiencies in hydrolysates. The fermentative performance of the new yeast strain and the co-utilization of mixed sugars can be further improved by strain adaptation to nutritionally deficient conditions and genetic engineering.

## Methods

### Microorganism and culture conditions

Soil samples were collected from Sri Chamarajendra Zoological Gardens, Mysore, India and stored in sterile containers at 4 °C until subsequent use. Approximately, 1 g of soil sample was inoculated into yeast extract peptone dextrose broth (YEPD) and incubated at 32 °C for 48 h. On obtaining growth, the liquid culture was serially diluted in sterile saline and plated onto agar plates with 1 % CMC which were incubated at 32 °C for 48 h. The colonies obtained were sub-cultured till pure isolates were obtained. Upon microscopic observations, one of the isolated colonies was found to be a yeast. The isolate was maintained on YEPD agar slants at 4 °C. Liquid cultures of the isolate were obtained by inoculating a loopful of culture from YEPD slants into YEPD broth and incubating at 32 °C for 24 h at 150 rpm.

### Identification of new strain

Strain identification of the new yeast isolate was done by sequencing the 18S, 5.8S and 26S rRNA and the isolate was found to be *C. tropicalis*. The primers used for strain identification spanned the 18S rRNA, the variable D1-D2 domain of the 26S rRNA and the internal transcribed spacer region of the 5.8S rRNA. The primer sequences were as follows: 18S_fwd 5′–TCCTCCGCTTATTGATATGC-3′, 18S_rev 5′-GAAGTAAAAGTCGTAACAAGG-3′,26S_fwd 5′GCATATCAATAAGCGGAGGAAAAAG-3′, 26S_rev 5′-GGTCCGTGTTTCAAGACG-3′, ITS1 5′TCCGTAGGTGAACCTGCGG-3′ and ITS4 5′-TCCTCCGCTTATTGATATGC-3′. The PCR conditions used were: initial denaturation at 95 °C for 5 min, followed by 30 cycles of denaturation, annealing and extension at 95, 52 and 72 °C, respectively, for 30 s or 1 min. A final product extension at 72 °C for ten minutes was also done before analysing the PCR products by gel electrophoresis. The PCR products were purified using the GeneJET gel extraction kit (Thermo Fisher Scientific) before sequencing. The strain was submitted to Microbial Type Culture Collection (MTCC), India and assigned the accession number -MTCC 25057.

### Biomass pretreatment and enzymatic hydrolysis

Wheat straw procured from local sources around Bangalore was stored and used throughout the study. Wheat straw was subjected to phosphoric acid (0.2 % v/v) pretreatment as described previously [63]. The cellulose, hemicellulose and lignin composition of the wheat straw was determined using standard NREL procedures [64]. The liquid hydrolysate obtained was neutralized to pH 6–7 using ammonia and used for cellulolytic enzyme production. The solid biomass obtained after phosphoric acid pretreatment was washed with distilled water, dried at 60 °C and used for enzymatic hydrolysis. Briefly, 5 g of pretreated biomass, 20 U g^−1^ ds enzyme and 50 mL sodium citrate buffer (50 mM, pH 4.8) were taken in 250 mL Erlenmeyer flasks and incubated at 50 °C and 150 rpm shaking for 24–48 h. The sugar hydrolysate was separated by centrifugation at 15,000 rpm for 10 min and the reducing sugar content was determined by uHPLC.

### Cellulase and xylanase production under submerged fermentation

Submerged fermentation was carried out by inoculating 0.2 mL of the exponential phase MTCC 25057 culture to 250 mL Erlenmeyer flasks containing 20 mL of wheat straw hydrolysate supplemented with minimal salts. The composition of minimal salts (g L^−1^) was—NaNO_3_: 2.5, KH_2_PO_4_: 1, MgSO_4_·7H_2_O: 0.5, KCl: 0.5. To these flasks, cellulosic substrates (CMC or pretreated WS) at different concentrations (0.5 % w/v, 1 % w/v and 2 % w/v) were added. The cultures were incubated on a rotary shaker (150 rpm) at different temperatures (32 and 42 °C) and for varying periods (24, 48, 72 and 96 h). The crude supernatant obtained by centrifuging the culture broth at 8000 rpm for 10 min was used for determining enzyme activity.

### Enzyme assays

Crude endoglucanase activity was determined by the CMC method [65]. The substrate was prepared by solubilizing carboxymethyl cellulose (Sigma) (2 % w/v) in 0.05 M sodium citrate buffer at pH 4.8. Briefly, 0.5 mL of the crude enzyme supernatant (diluted appropriately) was added to 0.5 mL of the substrate and incubated at 50 °C for 30 min. The reducing sugars liberated were estimated by the DNS method [66]. Crude exoglucanase activity was determined by the filter paper assay method [67]. The substrate used was 50 mg of Whatman No. 1 filter paper in 0.05 M sodium citrate buffer at pH 4.8. The crude enzyme supernatant (0.5 mL) was added to 1 mL of the buffer having 50 mg filter paper and incubated at 50 °C for 60 min and the reducing sugars liberated were estimated by the DNS method. Xylanase activity was determined by the beechwood xylan assay method [65]. Substrate preparation was done by adding beechwood xylan (Sigma) (1 % w/v) to 0.05 M sodium citrate buffer, pH 4.8. The appropriately diluted crude enzyme supernatant (0.1 mL) was added to 0.5 mL of the substrate and 0.4 mL of buffer and incubated at 50 °C for 15 min. The reducing sugars released were measured by the DNS method. All the estimations were carried out in triplicates. The enzyme activity was expressed in terms of International units (IU) for endoglucanase and xylanase and filter paper unit (FPU) for exoglucanase, respectively, or as units per gram of dry substrate (gds), i.e., U g^−1^ ds. One IU or FPU is defined as the amount of enzyme activity required to release one micromole of reducing sugar from a cellulosic substrate (CMC, xylan, filter paper, etc.) per mL in a minute under the suitable assay conditions.

### Fermentation conditions

The *C. tropicalis* MTCC 25057 strain was grown in 50 mL of yeast extract—peptone broth (20 g L^−1^ peptone and 10 g L^−1^ yeast extract) supplemented with either 10 % glucose, 10 % xylose or a mixture of 5 % glucose and 5 % xylose in 250 mL Erlenmeyer flasks in an incubator shaker maintained at 32 °C and 150 rpm. Samples were collected at appropriate intervals and the metabolites produced were analysed using UHPLC. Anaerobic condition was maintained by flushing nitrogen initially through the headspace of the flask and then incubating at 32 °C as mentioned previously. For inhibitor tolerance studies, furfural, 5-hydroxymethylfurfural or acetic acid (Himedia Laboratories) was added to the yeast extract—peptone broth with 1.5 % xylose and 0.5 % glucose (to simulate pretreated hydrolysate), in concentrations ranging from 0.5 to 3 g L^−1^ and grown at 32 °C for 24 h. The yeast was grown in YEPD, i.e., yeast extract peptone broth with 20 g L^−1^ glucose medium maintained at different pH (4, 5, 6, 7, 8, 9, 10) and at different temperatures (32, 37, 42, 45, 50 °C) for 24 h for determining the pH and temperature tolerance of the strain.

The pretreated and sugar hydrolysates were supplemented either with minimal salts (as mentioned previously) or with 10 g L^−1^ yeast extract for ethanol/xylitol production studies. The yeast was inoculated from a fresh plate into 50 mL hydrolysate and incubated at 150 rpm at 32 °C in 250 mL Erlenmeyer flasks. The samples were collected at suitable intervals and analysed.

### Analytical methods

The soluble sugars or metabolites content in the culture samples were determined using a 1290 Infinity series UHPLC system (Agilent) equipped with a Hiplex H anion exchange column (Agilent). Filtered and degassed 5 mM H_2_SO_4_ was used as the mobile phase at a flow rate of 0.6 mL/min. The column was maintained at 45 °C in a thermostat chamber, while the refractive index (RI) detector was maintained at 55 °C. The concentrations of glucose, xylose, ethanol and xylitol were estimated using appropriate calibration curves. The biomass or cell density of the culture samples was estimated by measuring the optical density at 600 nm using a Perkin Elmer Lambda 35 UV/visible spectrophotometer.

## Abbreviations

MTCC: microbial type culture collection
CBP: consolidated bioprocessing
HMF: hydroxymethylfurfural
CMC: carboxymethylcellulose
WS: wheat straw
YEPD: yeast extract peptone dextrose
NADPH: nicotinamide adenine dinucleotide phosphate
N: nitrogen
SNAREs: soluble NSF Attachment Protein Receptor
PCR: polymerase chain reaction
NREL: National Renewable Energy Laboratory
DNS: dinitrosalicylic acid
UHPLC: ultra high pressure liquid chromatography
RI: refractive index
